# The influence of host genotype and gut microbial interactions on feed efficiency traits in pigs

**DOI:** 10.3389/fmicb.2024.1459773

**Published:** 2024-11-13

**Authors:** Zhuoda Lu, Tao Zhang, Yunxiang Zhao, Yanqin Pang, Meng Guo, Xiaoping Zhu, Ying Li, Zhili Li

**Affiliations:** ^1^School of Animal Science and Technology, Foshan University, Foshan, China; ^2^Guangxi Yangxiang Co., Ltd., Guigang, China; ^3^College of Animal Science and Technology, Guangxi University, Nanning, China

**Keywords:** pig, gut microbial, genome-wide association analysis, growth performance, feed efficiency, genetic variation pig, genetic variation

## Abstract

Feed efficiency and growth performance are economically important traits in pigs. Precious studies have been revealed that both genetics and gut microbes could influence host phenotypes, however, the mechanisms by which they affect pig growth and feed efficiency remain poorly understood. In this study, 361 crossbred Duroc × (Landrace × Yorkshire) commercial pigs were genotyped using GeneSeek Porcine SNP50K BeadChip, and the microbiotas from fecal samples were acquired using microbial 16S rRNA gene sequencing technology to investigate the impact of host genetics and gut microorganisms on growth and feed efficiency. The results showed that the heritability and enterobacterial force ranged from 0.27 to 0.46 and 0 to 0.03, respectively. Genome-wide association studies (GWAS) identified seven significant SNPs to be associated with growth and feed efficiency, and several genes, including *AIF1L*, *ASS1*, and *QRFP* were highlighted as candidates for the analyzed traits. Additionally, microbiome-genome-wide association studies GWAS revealed potential links between *CCAR2*, *EGR3*, *GSTM3*, and *GPR61* genes and the abundance of microorganisms, such as *Trueperella*, *Victivallis*, and *Erysipelatoclostridium*. In addition, six microbial genera linked to growth and feed efficiency were identified as follows *Lachnospiraceae_UCG-005*, *Prevotellaceae_UCG-003*, *Prevotellaceae_NK3B31_group*, *Prevotella_1*, *Prevotella_9*, and *Veillonella*. Our findings provide novel insights into the factors influencing host phenotypic complexity and identify potential microbial targets for enhancing pig feed efficiency through selective breeding. This could aid in the development of strategies to manipulate the gut microbiota to optimize growth rates and feed efficiency in pig breeding.

## 1 Introduction

With the development of intensive pig farming, feed costs have become the main expense in raising livestock (Si et al., 2020). This transformation has made improving the growth rate and feed efficiency of pigs the core focus of breeding work. The feed conversion ratio (FCR) and residual feed intake (RFI) are two key traits used to assess feed efficiency (Davoudi et al., 2022). Significant genetic correlations have been demonstrated between these traits and average daily gain (ADG) as well as average daily feed intake (ADFI) (Do et al., 2013; Herrera-Cáceres et al., 2020), establishing them as critical indicators of feeding efficacy. Moreover, GWAS has been shown to be an effective approach for identifying loci associated with feed efficiency and related quantitative trait loci (QTL), along with candidate genes in swine (Do et al., 2014; Horodyska et al., 2017; Ding et al., 2018). Numerous single-nucleotide polymorphisms (SNPs) have been identified on SSC1, SSC4, SSC6, SSC7, and SSCX as being significantly correlated with FCR. Notably, the marker WU_10.2_7_18377044 on SSC7 is estimated to account for approximately 2.37% of the phenotypic variance in RFI; additionally, DRGA0001676 on SSC 1 has been shown to explain 3.22 and 5.46% of the phenotypic variance in FCR and RFI, respectively (Ding et al., 2018). These findings are expected to facilitate advancements in genomic selection for both feed efficiency and its associated traits.

As scientific research deepens, the function of intestinal flora has attracted increasing attention. Tang et al. (2020) through 16S rRNA gene sequencing of microbial samples from different intestinal segments, found that the microbiota in the cecum and colon significantly affects body weight (BW) and ADG. Hu et al. (2018) conducted a study where they fed fecal microbiota suspensions from healthy adult Jinhua pigs to Duroc × (Landrace × Yorkshire) (DLY) piglets. The results indicated that piglets receiving fecal microbiota transplantation (FMT) showed increased ADG and a significantly lower incidence of diarrhea. This discovery underscores that exogenous FMT can modulate gut microbiota composition and positively influence growth performance, gut barrier function, and innate immunity in animals. It further confirms the critical role of gut microbiota in animal growth and health.

The gut microbiota of pigs is considered a crucial “organ” that plays a central role in nutrient processing and energy intake (Fouhse et al., 2016; Ramayo-Caldas et al., 2016; Xiao et al., 2016). Quan et al. (2018) research indicates that pigs with high FCR have gut microbiota in the cecum and colon that may process dietary polysaccharides and proteins more efficiently than those with low FCR. These microbes produce short-chain fatty acids (SCFAs) and indole compounds during fermentation, which help improve feed efficiency and promote gut health in pigs. Several studies have shown that certain bacteria associated with higher feed efficiency (FE) can ferment various substrates, particularly those related to butyrate production. These bacteria primarily include *Ruminococcaceae* in the cecum and colon, as well as *Butyricicoccu*s in the cecum and colon (Holman et al., 2017; Tan et al., 2017; Quan et al., 2018; Quan et al., 2020; Vigors et al., 2020). However, the relationship between host genetics and gut microbiota in relation to growth and feed efficiency remains poorly understood. A few recent studies have suggested that a significant portion of the variability in the gut microbial community is genetically controlled and has genetic links to feed efficiency (Aliakbari et al., 2021). Similarly, several studies in chickens have identified heritable microbiota associated with feed efficiency (Wen et al., 2021; Zhang et al., 2024). Nevertheless, it remains largely unclear whether host genetics influence feed efficiency by promoting a stable gut microbial community. Evidence from GWAS has identified several host genetic variations that affect gut microbiota. For instance, 50 single-nucleotide polymorphisms were found to be associated with two microbial taxa in chickens. Li et al. (2019) reported that 19 SNPs were associated with 14 rumen microbial taxa in cattle. However, potential host genotypes related to gut microbiota in pigs have not been well characterized. Recently, Wang Y. et al. (2022) reported the effect of host genetics and the gut microbiome on pig fat deposition traits, suggesting the possibility that host genetic variation may shape gut microbial composition in pigs.

The gut microbiota of pigs, often referred to as the “second genome”, plays a crucial role in the host nutrient digestion, energy intake, and disease resistance. Despite previous research (Bergamaschi et al., 2020a; Quan et al., 2020; Tang et al., 2020; Aliakbari et al., 2022; Déru et al., 2022; Wang Y. et al., 2022), the mechanisms by which host genetics influence gut microbiota and interact with it to affect pig production and feed efficiency traits remain unclear. Understanding the relationship between host genetics and gut microbiota in production and feed utilization is essential for developing effective strategies to enhance production and improve feed efficiency. We used the GeneSeek Porcine SNP50K BeadChip to genotype 361 DLY pigs and obtained their microbiotas from fecal samples using 16S rRNA gene sequencing technology. Our aim was to screen for differential bacterial genera affecting feed efficiency and growth performance and to explore the mutual influence of host genotype and gut microbiota on host phenotype. This could help devise strategies to manipulate the gut microbiota, aiming to enhance growth rates and feed efficiency in pig breeding.

## 2 Materials and methods

### 2.1 Animals, phenotypes, and sample collection

Three hundred and sixty one DLY commercial pigs (187 males and 174 females) were purchased from a pig farm in Guangxi. Male DLY piglets were castrated on the 6th to 7th day after birth. All pigs were raised under the same management conditions with free access to water and feed. An electronic feeding station was utilized to collect raw data, including pig weight and feed intake per feeding. In this study, electronic feeding stations were used to record the raw data of pigs’ body weight and feed intake from 30 to 100 kg. Pigs were slaughtered in the same commercial abattoir at 113 ± 5 days of age. Rectal contents were collected from pigs prior to slaughter. All samples were immediately frozen in liquid nitrogen and stored at −80°C until further processing.

ADFI, ADG, and FCR (Casey et al., 2005; Jiao et al., 2014) were calculated by quality-controlling the raw data on body weight, feed intake, and time. RFI was calculated according to the method described by Do et al. (2014), using onset body weight (OnBW), ADG, and ADFI. The normality of all traits was assessed with the Shapiro–Wilk test in R (version 4.2.1). Final quality control of all phenotypic data was performed by excluding values beyond the mean ± 3 standard deviations.

### 2.2 Genotyping and quality control

DNA was extracted from ear tissue using the Genome Extraction Kit (Wuhan NanoMagBio Technology Co., Ltd., China). The quality of the DNA was assessed by measuring the optical absorption ratios (A260/280 and A260/230) to ensure concentrations ≥40 ng/μl. Genotyping of the genomic DNA was performed using the GeneSeek Porcine 50 K SNP Beadchip (GeneSeek, Lansing, MI, United States). Genotype imputation for missing data points was conducted using Beagle 5.2 (Browning et al., 2021). Quality control of the SNP data, both before and after imputation, was performed using PLINK software (Chang et al., 2015). The quality control parameters were as follows: SNP call rate > 0.95, minor allele frequency > 0.01, and individual genotype call rate > 0.95. After quality control, 43,580 SNPs per pig were retained for further analysis.

### 2.3 16S rRNA gene sequencing and analysis

Genomic DNA from fecal microbiota was extracted using the cetyltrimethylammonium bromide (CTAB) method. The V4-V5 region of the 16S rRNA gene was amplified using barcoded primers 515F/907R. All PCR reactions were performed with the Phusion^®^ High-Fidelity PCR Master Mix with GC Buffer from New England Biolabs. The forward primer used was 515F (5′-GTGCCAGCMGCCGCGGTAA-3′) and the reverse primer was 907R (5′-CCGTCAATTCCTTTGAGTTT-3′). To ensure amplification efficiency and accuracy, we used high-efficiency, high-fidelity enzymes for the PCR. The PCR was carried out on a Bio-Rad T100 gradient PCR machine. The PCR products were checked by electrophoresis on a 2% agarose gel. Qualifying PCR products were purified using the GeneJET Gel Extraction Kit (Thermo Scientific, United States). Library preparation was performed using the TruSeq DNA PCR-Free Library Preparation Kit (Illumina). The constructed libraries were quantified using Qubit and assessed for quality. Sequencing was carried out on the Illumina NovaSeq 6000 platform, with amplicon libraries sequenced on the Illumina MiSeq 2 × 250 platform, provided by Novogene (Beijing, China). Each sample generated approximately 95,354 clean reads. Bioinformatics analysis of the amplicon sequencing was conducted using EasyAmplicon v1.0 (Liu et al., 2021). Paired-end sequence data were merged, quality filtered, and dereplicated using VSEARCH v2.15 (Rognes et al., 2016) subcommands-fastq_mergepairs, −fastx_filter, and-derep_fulllength, respectively. Non-redundant sequences were then denoised into Operational Taxonomic Units (OTUs) using USEARCH v10.0 (Edgar, 2010). Chimeras were removed using VSEARCH-uchime_ref against the SLIVA (Quast et al., 2013) database. A feature table was created with VSEARCH-usearch_global, and species annotation was performed using the Greengenes (DeSantis et al., 2006) database gg_16s_13.5 sequences with the USEARCH-sintax algorithm. Diversity analysis was conducted using the vegan v2.4–6 package, and visualizations were generated with ggplot2 v3.5.0. Linear discriminate analysis effect size (LEfSe) analysis was performed through the online platform ImageGP (Chen et al., 2022), the LEfSe’s threshold on the logarithmic score of LDA was set to 2.0, the remaining settings were default parameters. The functional profiles of microbial communities were predicted using PICRUSt (Langille et al., 2013) with the Greengenes database as the reference.

### 2.4 Construction of host genetic relationship matrix and microbial relationship matrix

Through quality control, a total of 43,580 SNPs were selected for principal component analysis (PCA) and the construction of the genetic relationship matrix (GRM) using GCTA (ver 1.91.1) (Yang et al., 2011):


$$g_{ij}=\frac{1}{N}\overset{N}{\underset{v=1}{\sum }}\frac{\left(x_{iv}-2\bar{p_{v}}\right)\left(x_{jv}-2\bar{p_{v}}\right)}{2\bar{p_{v}}\left(1-\bar{p_{v}}\right)}$$

Here, $g_{ij}$ represents the estimated genetic relationship between DLY individuals *i* and *j*; $x_{iv}$ and $x_{jv}$ are the counts of the reference allele for individuals *i* and *j* respectively; $\bar{p_{v}}$ is the frequency of the reference allele in the population; and *N* is the number of variant sites.

We construct the microbial relationship matrix (MRM) based on the relative abundance of OTU using R script, the formula is $M={XX}^{T}\left(\frac{1}{p}\right)$ , with matrux X and *p* is the number of OTUs (Camarinha-Silva et al., 2017). Each element of matrix P is the relative abundance of OTU*j* in animal *i* (plus 1). Matrix P is used to compute matrix X, which is calculated as follows:


$$X_{ij}=\frac{\log\left(P_{ij}\right)-\log\bar{P_{j}}}{sd\left(\log P_{j}\right)}$$

Where *Pj* is the *j* column vector of the matrix *P*.

### 2.5 Interaction between host genetics and gut microbiota

The following multiple random effects model was established to estimate variance components of the target traits using HIBLUP software (Wang Y. et al., 2022; Yin et al., 2023).


$$y=1\mu +Z_{1g}+Z_{2m}+Z_{3a}+e$$

*y* is the vector of phenotypes (ADG, ADFI, FCR, RFI); μ is the overall mean; $g\sim N\;\left(0,G\sigma_{g}^{2}\right)$ is the vector of host genetic random effect, where *G* and $\sigma_{g}^{2}$ are the GRM and host genetic variance; $m\sim N\;\left(0,M\sigma_{m}^{2}\right)$ is the vector of gut microbiome random effect, where *M* and $\sigma_{m}^{2}$ are the MRM and gut microbiome variance; $a\sim N\;\left(0,A\sigma_{a}^{2}\right)$ is the vector of interactions between host genetics and gut microbiome random effect, where *A* and $\sigma_{a}^{2}$ are the GRM×MRM and variance of interactions between host genetics and gut microbiome; e is the residual effect; $Z_{1}$ , $Z_{2}$ , $Z_{3}$ are, respectively, the corresponding incidence matrices of *g*, *m*, and *a*.

### 2.6 Estimating the effects of host genetics and gut microbiota on growth and feed efficiency

Since the D (LY) commercial pigs used in this study lack pedigree information, the heritability of the target traits was estimated based on SNPs using the following model:


$$y=Kc+g+e\left[A\right]$$

*y* is the vector of phenotypes (ADG, ADFI, FCR, RFI); *c* is the vector of fixed covariates, including the effects of sex (2 level), pen (2 level), initial body weight (81 level) (Xiang et al., 2024), and the first three principal components of host genetics; *K* is the matrix corresponding to *c*; *g* is the vector of total SNP effects, which follows *a* ∼ *N* (0, $G\sigma_{A}^{2}$ ), where *G* is the host genetic relationship matrix (GRM) and $G\sigma_{A}^{2}$ is the polygenic genetic variance; e is the residual effect. Heritability is defined as $h^{2}=\frac{\sigma_{g}^{2}}{\sigma_{p}^{2}}$ , where $\sigma_{p}^{2}$ is the phenotypic variance. Additionally, we included microorganisms with a detection rate greater than 60% in the sample as a quantitative trait, while those present in 30–60% of the samples were treated as binary traits. Microorganisms detected in less than 30% of the samples were excluded from the analysis (Zierer et al., 2018).

Microbiability, the proportion of phenotypic variance explained by gut microbiota variance, is defined as: $m^{2}=\frac{\sigma_{m}^{2}}{\sigma_{p}^{2}}$ , where $\sigma_{m}^{2}$ is the microbial variance and $\sigma_{p}^{2}$ is the phenotypic variance. Microbiability ( $m^{2}$ ) was estimated using the following model in GCTA (ver 1.91.1):


$$y=Kc+m+e\left[B\right]$$

In which *y*, *K*, *c*, and *e* are defined the same as in the previous model [A]. *m* represents the gut microbiota effect, which follows a multivariate normal distribution $m\sim N\left(0,M\sigma_{m}^{2}\right)$ is the microbial relationship matrix (MRM) calculated using the aforementioned formula. We used the MRM in place of the GRM to estimate $m^{2}$ with GCTA.

Microbial genera with significant heritability were also included in the scope of investigation. Thus, we further performed GWAS analysis to detect significant host genetic markers affecting the phenotypes and microbial genera using the following linear mixed model in GEMMA (ver 0.98.1) (Zhou and Stephens, 2012):


$$y=Q\alpha +Χ\beta +g+e\left[C\right]$$

In which y is the vector of individual phenotypes (ADG, ADFI, FCR, RFI, the abundance or presence/absence of heritable genera); *Q* is the matrix of covariates, including sex (2 level), pen (2 level), initial body weight (81 level), and the first three principal components of host genetics; *α* is the vector of covariate effects, including the intercept; *X* is the vector of allele counts (0, 1, 2); *β* is the SNP effect. *g* is the vector of polygenic effects following a normal distribution *N* (0, $G\sigma_{g}^{2}$ ), where *G* is the genetic relationship matrix calculated from genome-wide marker information and $\sigma_{g}^{2}$ is the polygenic additive variance. *e* is the residual effect. To reduce the number of false negatives, the FDR method was used to determine the significance threshold, defined as $P=0.01\times \left(n/i\right)$ , where *n* represents the number of SNPs in the GEMMA-based GWAS results with *p* < 0.01, and i is the total number of eligible SNPs.

### 2.7 Identification of specific microbiota associated with growth and feed efficiency

Due to the low information content of taxa with low detection rates for association analysis, we only retained taxa that appeared in more than 30% of the specific sample types. The association analysis between qualifying taxa and growth and feed efficiency traits was conducted using a two-part model, as described by Fu et al. (2015) with a custom R script. This model considers both binary traits (presence and absence) and quantitative traits, as detailed below:


$$y=\left\{\frac{\beta_{1}b+e}{\beta_{2}q+e}\right\}$$

In this model, *y* represents the phenotypic value, $\beta_{1}$ denotes the estimated effect of the binary model, b is the binary trait, and e is the residual. For quantitative analysis, the model $y=\beta_{2}q+e$ was used to test the association between microbial abundance and phenotype. *p*-values were obtained from the two-part model association analysis. If the *p*-value from the binary model is less than 0.05, the presence or absence of the microorganism is considered to influence the phenotype. If the *p*-value from the quantitative model is less than 0.05, the phenotype is considered to be associated with the relative abundance of the microorganism. If both models have *p*-values less than 0.05, the phenotype is considered to be associated with both the presence/absence and the relative abundance of the microorganism.

To identify specific microbes that significantly impact the phenotypes under study, we performed an analysis of variance (ANOVA) to test for phenotypic differences between pigs with the highest (*N* = 36) and lowest (*N* = 36) abundances of a given microbe. Additionally, a Wilcoxon rank-sum test was conducted to determine the relative abundance differences of each taxon between pigs with the highest (*N* = 36) and lowest (*N* = 36) phenotypic rankings. A microbe was considered significant if the adjusted *p*-values from the two-part model association analysis, ANOVA, and Wilcoxon rank-sum test were all less than 0.05. Furthermore, we calculated the Pearson correlation between phenotypes and microbial genera using the psych package in R (|*r*| > 0.1). A correlation was deemed significant if the *p*-value was less than 0.05.

## 3 Results

### 3.1 Descriptive statistics of host phenotypes with genotypic sequencing results

Table 1 presents the descriptive statistics for host feed efficiency-related traits. In all the phenotypes studied, the coefficient of variation was below 15%. The correlation between ADG and RFI was negligible, but FCR and RFI showed a strong phenotypic correlation (*r* = 0.79, *p* < 0.001) (Supplementary Figure S1).

**Table 1:** Summary of feed efficiency traits in the resource population.

Trait	*N*	Mean	SD	CV (%)	Max	Min
RFI	361	0.36	0.16	–	0.89	−0.09
FCR	361	2.48	0.22	9.02	3.71	1.88
ADFI (Kg/d)	361	2.34	0.24	10.32	3.10	1.63
ADG (Kg)	361	0.95	0.10	10.93	1.38	0.50

N is the number of non-missing values; SD is standard deviation; CV is variation coefficient; RFI is the residual feed intake; FCR is the feed conversion ratio; ADFI is the average daily feed intake; ADG is the average daily gain.

### 3.2 Fecal microbial sequencing results and correlation analysis

The analysis of fecal microbiota data revealed a total of 36,320,886 sequences, with an average of 96,087 sequences per sample. These sequences were clustered into 3,568 OTUs at a 97% similarity threshold, encompassing 50 phyla, 99 classes, 194 orders, 326 families, and 699 genera.

**Figure 1:**
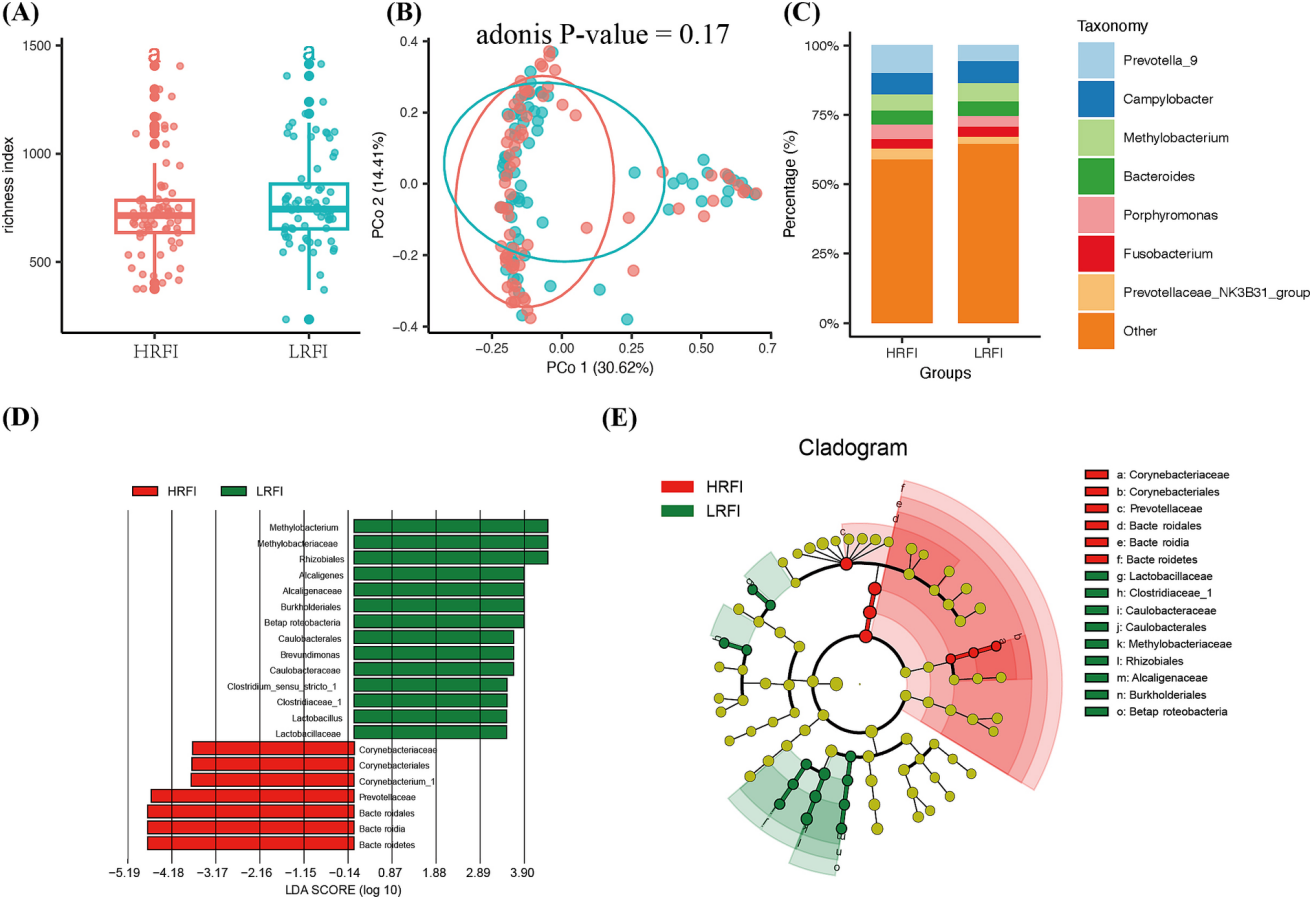
The microbiota composition between the high RFI and low RFI groups. (A) The *α*-diversity index richness compared between the two groups. (B) The Principal Coordinates Analysis at the OTU level. (C) Composition of fecal microbiota in the two groups. (D,E) LEfSe analysis results for high and low RFI groups.

In this study, we ranked the phenotypic values of RFI and selected the top and bottom 20% to form high (HRFI) and low (LRFI) groups for differential analysis from a gut microbiota perspective. The species richness in both HRFI and LRFI groups plateaued at around 700, indicating adequate sample detection rates (Figure 1A). PCoA analysis using the Bray-Curtis distance method showed no significant difference in beta diversity between the high and low RFI groups (*p* > 0.05, Figure 1B). The stacked bar chart of the relative abundance of dominant genera at the genus level showed that the top seven dominant genera were *Prevotella_9*, *Campylobacter*, *Methylobacterium*, *Bacteroides*, *Porphyromonas*, *Fusobacterium*, and *Prevotellaceae_NK3B31_group* (Figure 1C). LEfSe analysis of microbial differences between phenotypic groups revealed that at the genus level (Figures 1D,E), *Bacteroides* was mainly enriched in the HRFI, while *Clostridium*, *Lactobacillus*, *Methylobacterium*, *Methanobacterium*, and *Alcaligenes* were enriched in the LRFI, with *Bacteroides* having the highest score.

### 3.3 Influence of host genotype and gut microbial interactions on traits related to growth performance and feed efficiency in pigs

Table 2 shows that ADG is independently influenced by host genetic effects and microbiome effects, while the other three traits are influenced by host genetic effects, microbiome effects, and their interactions. The independent effects of host genetics and gut microbiota on ADG were 0.37 and 0.12, respectively. Previous studies have shown that, considering other environmental factors, host genetic effects are higher than gut microbiota effects, which is consistent with our findings. ADFI and FCR exhibited genetic effects of 0.31 and 0.19, microbiome effects of 0.01 and 0.02, and host genetic and gut microbiota interaction effects of 0.26 and 0.15, respectively. Notably, the interaction effect for RFI was 0.5, the highest among all traits, indicating that the interaction between host genotype and gut microbiome may play a crucial role in improving feed efficiency.

**Table 2:** Host genetics and gut microbiome effects for feed efficiency traits.

Trait	*g*			*m*			*a*		
	$\sigma_{g}^{2}$	$\sigma_{g}^{2}/\sigma_{p}^{2}$	*p*-value	$\sigma_{m}^{2}$	$\sigma_{m}^{2}/\sigma_{p}^{2}$	*p*-value	$\sigma_{a}^{2}$	$\sigma_{a}^{2}/\sigma_{p}^{2}$	*p*-value
RFI	0.0030	0.2007	0.3562	0.0007	0.0304	0.3920	0.0117	0.5087	0.0465
FCR	0.0076	0.1949	0.0847	0.0009	0.0231	0.5182	0.0058	0.1487	0.4709
ADFI	0.015	0.3125	0.01446	0.0006	0.0125	0.6281	0.0123	0.2562	0.2342
ADG	0.0048	0.3692	0.0006	0.0015	0.1154	0.1641	–	–	–

g, the effect of host genetics; m, the effect of gut microbiome; a, the effect of interactions between host genetics and gut microbiome; RFI residual feed intake; FCR feed conversion ratio; ADFI average daily feed intake; ADG average daily gain. The significant *p*-value bounds are *p* < 0.05.

### 3.4 Heritability and enterobacterial power of traits related to feed efficiency

**Figure 2:**
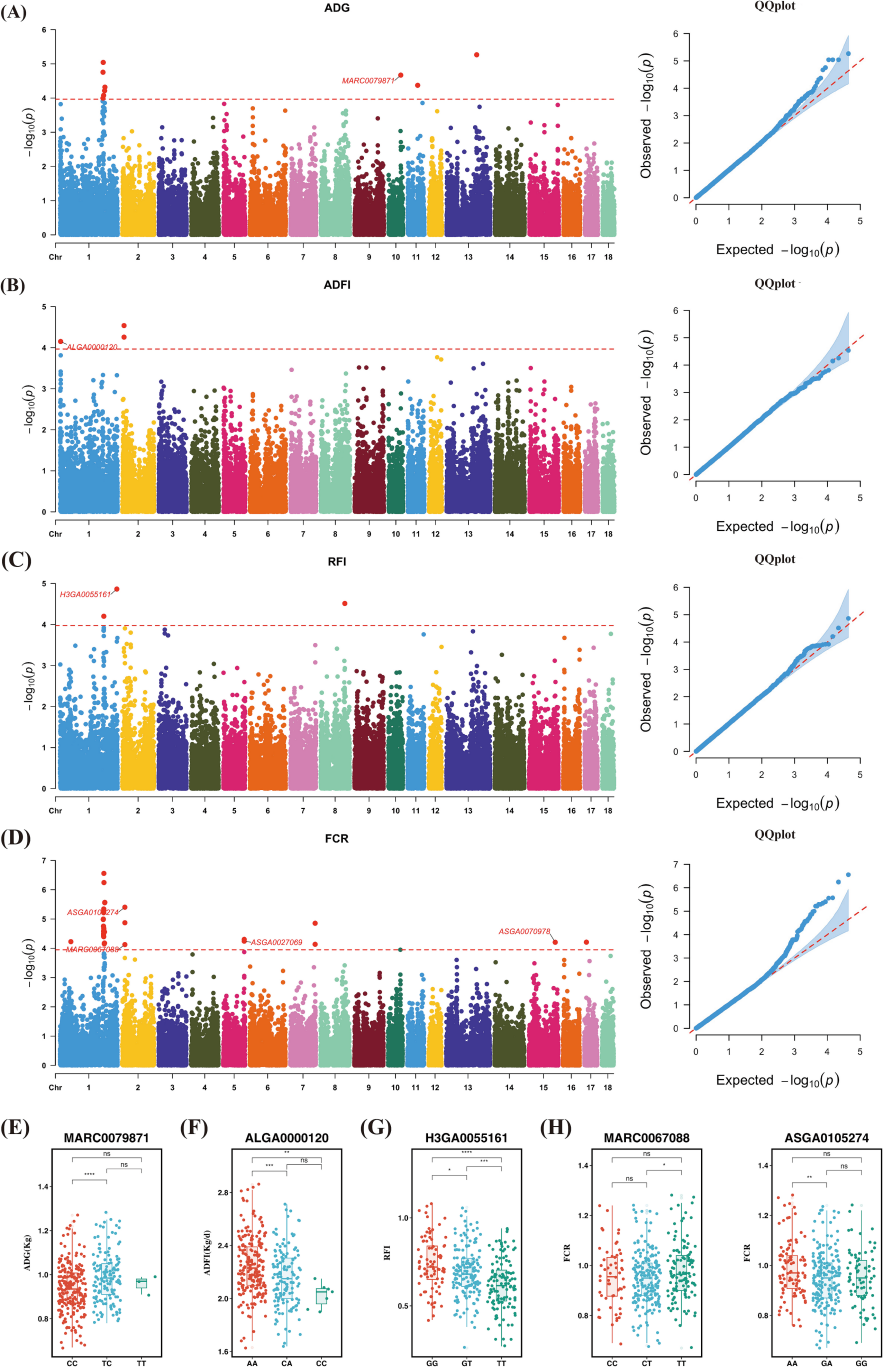
Genome-wide association analysis. (A,E) GWAS results of ADG and distribution of significant SNPs in different genotypes. (B,F) GWAS results of ADFI and distribution of significant SNPs in different genotypes. (C,G) GWAS results of RFI and distribution of significant SNPs in different genotypes. (D,H) GWAS results of FCR and distribution of significant SNPs in different genotypes.

In this study, the SNP-based heritability estimates for feed efficiency traits ranged from 0.27 to 0.46, indicating medium to high heritability levels (Supplementary Table S6). This suggests that host genetics play a significant role in regulating feed efficiency. Consequently, we conducted GWAS for these traits. The phenotypic data conformed to a normal distribution, and the corresponding Manhattan and QQ diagrams are shown in Figure 2. We identified 48 significant SNPs, seven of which were associated with growth performance and feed efficiency (Supplementary Table S1). The SNP MARC0079871 was a significant locus for ADG, located near the genes Cyclin Y (*CCNY*) and Cullin 2 (*CUL2*), with pigs of the TT genotype having significantly higher average daily gain compared to the other two genotypes (Figures 2A,E). Regarding ADFI, the SNP ALGA0000120 was located near the genes C-C Motif Chemokine Receptor 6 (*CCR6*) and Ribosomal Protein S6 Kinase A2 (*RPS6KA2*). The variation at this locus was likely due to base inversion (A/C), with pigs of the predominant AA genotype showing higher average daily feed intake than the other two genotypes (Figures 2B,F). In RFI, the SNP H3GA0055161 was found near the genes Allograft Inflammatory Factor 1 Like (*AIF1L*), Argininosuccinate Synthase 1 (*ASS1*), and Pyroglutamylated RFamide Peptide (*QRFP*). Pigs with the TT genotype exhibited significantly higher feed efficiency compared to the other genotypes, with average RFIs of 0.62, 0.69, and 0.74 for TT, GT, and GG genotypes, respectively (Figures 2C,G). For FCR, four SNPs were found to be associated with this trait. ASGA0027069 and ASGA0070978 were located near the genes Acyl-CoA Synthetase Short Chain Family Member 3 (*ACSS3*), Adaptor Related Protein Complex 1 Subunit Sigma 3 (*AP1S3*), Cullin 3 (*CUL3*), and Mitochondrial Ribosomal Protein L44 (*MRPL44*). ASGA0105274 was located near the genes Glycine-N-Acyltransferase Like 2 (*GLYATL2*) and Lactate Dehydrogenase B (*LDHB*). Notably, ASGA0105274 and MARC0067088 were both located near the genes Olfactory Receptor Family 5 Subfamily B Member 21 (*OR5B21*), Olfactory Receptor Family 5 Subfamily B Member 3 (*OR5B3*), Olfactory Receptor Family 9 Subfamily I Member 1 (*OR9I1*), Olfactory Receptor Family 9 Subfamily Q Member 1 (*OR9Q1*), and Olfactory Receptor Family 9 Subfamily Q Member 2 (*OR9Q2*), all of which belong to the OR family, and showed strong associations. At the ASGA0105274 locus, pigs with the AA genotypes had significantly higher feed efficiency than those with the GA genotype. At the MARC0067088 locus, pigs with the TT genotypes had significantly higher feed efficiency than those with the CT genotype (Figures 2D,H).

### 3.5 Microbiability and identification of host genome variants associated with gut microbiota

**Figure 3:**
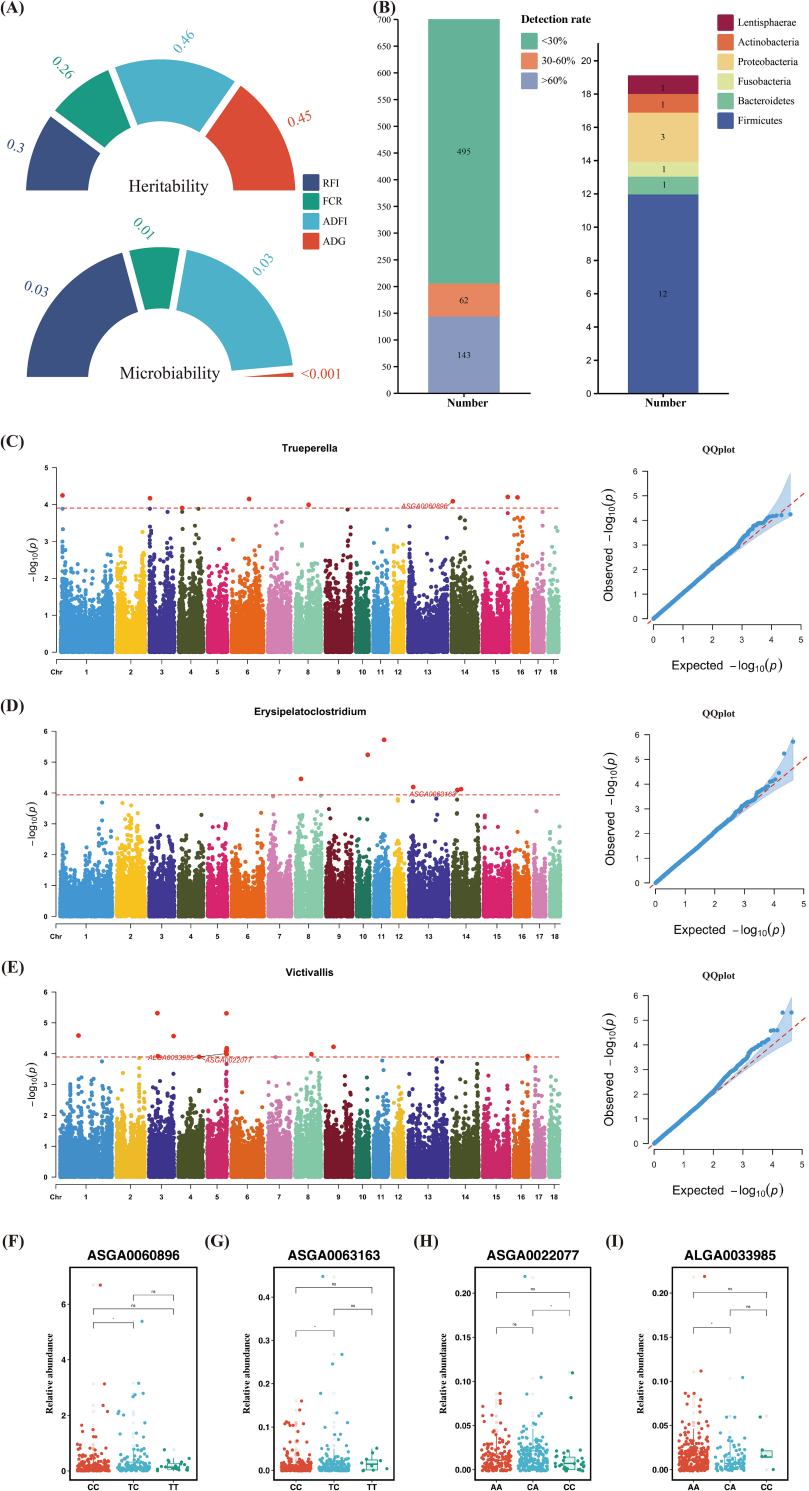
(A) Phenotypic heritability and microbiality. (B) Number of identified microbial genera and the number of significantly heritable microorganisms grouped by phyla. (C,F) Microbial genome-wide association studies results of *Trueperella* and distribution of significant SNPs in different genotypes. (D,G) Microbial genome-wide association studies results of *Erysipelatoclostridium* and distribution of significant SNPs in different genotypes. (E,H,I) Microbial genome-wide association studies results of *Victivallis* and distribution of significant.

Similar to heritability, microbiability is defined as the proportion of phenotypic variance attributable to microbial variance, reflecting the extent to which host phenotypes are influenced by gut microbiota. The $m^{2}$ for RFI, FCR, and ADFI were 0.03, 0.01, and 0.03, respectively, while the estimate for ADG was nearly zero (Figure 3A) (Supplementary Table S7). Although the microbiability of these traits is relatively low, host genetics may influence a small subset of low-abundance microbes that contribute minimally to the overall microbial community.

The results showed that we estimated the heritability for a total of 205 microbial genera, with 143 quantitative traits and 62 binary traits (Figure 3B). Heritability estimates revealed that 19 microbial genera had significant heritability (Figure 3B). Of the 205 microbial genera, we found that 13 in the Firmicutes, three in the Proteobacteria, and one each to Actinobacteria, Bacteroidetes, Fusobacteria, and Lentisphaerae, all exhibiting significant SNP-based heritability (*p* < 0.05) (Supplementary Table S8). Most of these heritable bacteria belonged to the Firmicutes and Proteobacteria phyla.

The mbGWAS identified 5, 11, and 6 significant genome-wide loci associated with *Trueperella*, *Victivallis*, and *Erysipelatoclostridium*, respectively (Supplementary Tables S2–S4). Genome-wide association analyses of the remaining bacterial genera are provided in Supplementary Figure S2. The most significant SNP controlling the relative abundance of *Trueperella* was ASGA0060896, located near the genes Cell Cycle And Apoptosis Regulator 2 (*CCAR2*) and Early Growth Response 3 (*EGR3*), with a minor allele frequency (MAF) of 0.24. Substitution of the TC genotype with CC at ASGA0060896 significantly increased the abundance of *Trueperella* (Figures 3C,F). For *Erysipelatoclostridium*, the SNP ASGA0063163 was identified as a locus controlling its relative abundance, with an MAF of 0.24. This SNP was located near the gene Phosphatidylinositol Transfer Protein Beta (*PITPNB*). There was a significant difference in the relative abundance of *Erysipelatoclostridium* between the TC and CC at this locus (Figures 3D,G). In *Victivallis*, the SNPs ASGA0022077 and ALGA0033985 were located near Glutathione S-Transferase Mu 3 (*GSTM3*), G Protein-Coupled Receptor 61 (*GPR61*), Colony Stimulating Factor 1 (*CSF1*), Adenosine Monophosphate Deaminase 2 (*AMPD2*), Myogenic Factor 5 (*MYF5*), Myogenic Factor 6 (*MYF6*), Protein Tyrosine Phosphatase Receptor Type (*PTPRQ*), and Acyl-CoA Synthetase Short Chain Family Member 3 (*ACSS3*), with MAFs of 0.36 and 0.12, respectively. At the ASGA0022077 locus, the CA genotype had a higher relative abundance of *Victivallis* compared to the CC genotype, while at the ALGA0033985 locus, the AA genotype had a higher relative abundance of *Victivallis* compared to the CA genotype, likely due to base inversion (A/C) (Figures 3E,H,I).

### 3.6 Fecal microorganisms associated with feed efficiency

**Figure 4:**
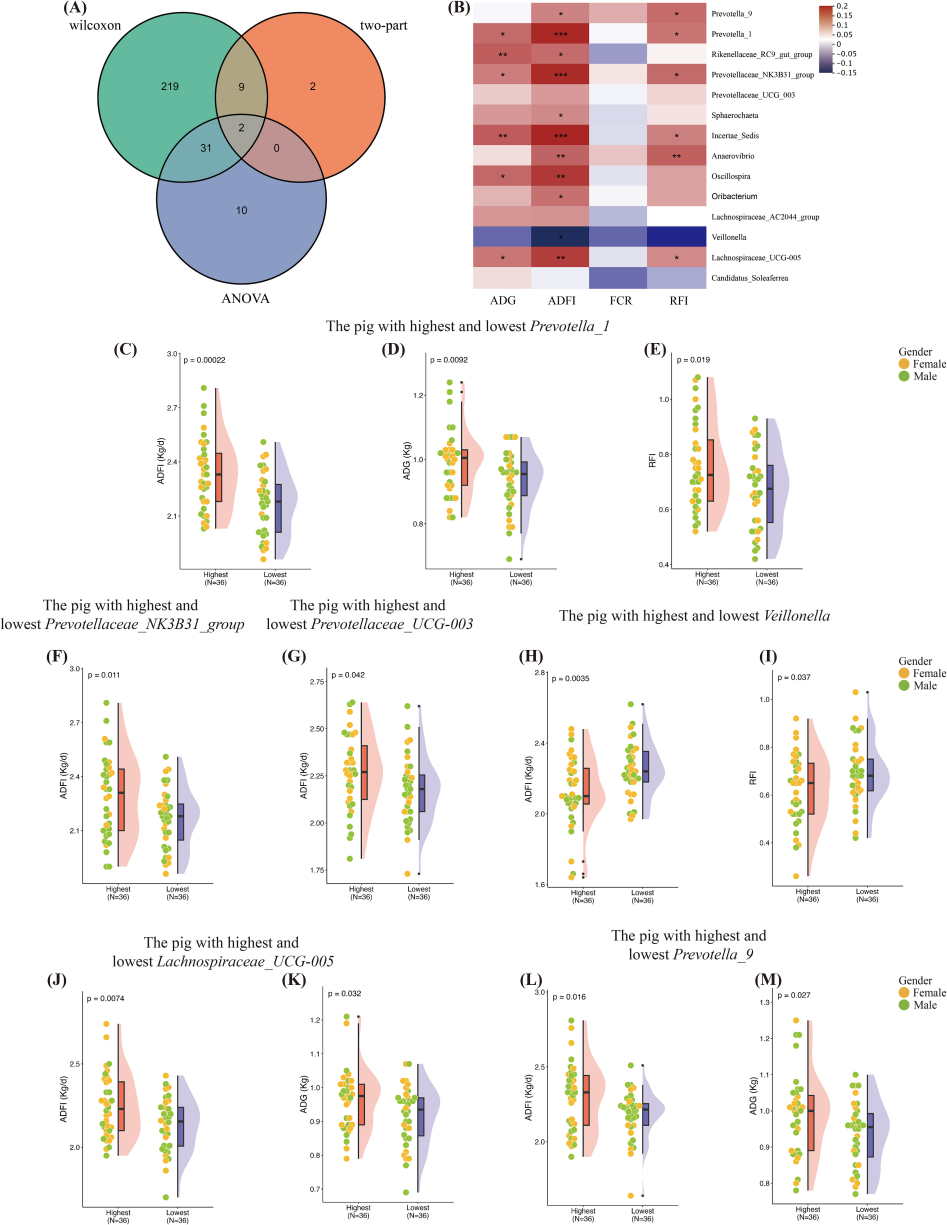
Identification of microbiota associated with growth and feed efficiency. (A) Number of microbial genera associated with phenotypic detected by each test or association analysis and their overlaps. (B) Pearson’s correlations among the shared 14 microbial genera. Significant *r* values are filled in with “*.” (C–E) Differences in the ADFI, ADG, RFI between the two groups with the highest and lowest *Prevotella_1* abundance. (F) Differences in the ADFI between the two groups with the highest and lowest *Prevotellaceae_NK3B31_group* abundance. (G) Differences in the ADFI between the two groups with the highest and lowest *Prevotellaceae_UCG_003* abundance. (H,I) Differences in the ADFI and RFI between the two groups with the highest and lowest *Veillonella* abundance. (J,K) Differences in the ADG, ADFI between the two groups with the highest and lowest *Lachnospiraceae_UCG-005* abundance. (L,M) Differences in the ADG, ADFI between the two groups with the highest and lowest *Prevotella_9* abundance.

Although host genetics can only influence a small portion of the gut microbiota, our findings indicate that both host genetics and gut microbiota can simultaneously affect feed efficiency in pigs. There may be a connection between the two, or a specific combination of microbes may be responsible for the observed effects. We conducted a two-part model association analysis and a two-tailed test for microbial genera and the traits under study. The two-part model analysis identified 13 associations. The Wilcoxon rank-sum test and ANOVA revealed 262 and 43 microbial genera, respectively. Among these analyses, 41 genera were consistently found in both the association analysis and the significance tests (Figure 4A) (Supplementary Table S5).

Given that more than half of the microbial genera had low detection rates, we focused on 14 genera with detection rates greater than 90% (Figure 4B). Pigs in the lowest 10% for *Prevotella_1* abundance had significantly lower ADFI, ADG, and RFI compared to those in the highest 10% (Figures 4C–E). Pigs in the highest 10% for *Prevotellaceae_NK3B31_group* and *Prevotellaceae_UCG_003* abundance had significantly higher ADFI than those in the lowest 10% (Figures 4F,G). Notably, pigs in the lowest 10% for *Veillonella* abundance had significantly higher ADFI and RFI than those in the highest 10% (Figures 4H,I). Pigs with higher abundance of *Lachnospiraceae_UCG-005* and *Prevotella_9* had higher ADFI and ADG than those with lower abundance (Figures 4J–M).

## 4 Discussion

In recent years, traits related to production and feed efficiency have had a significant impact on the sustainability of the pig farming industry due to their crucial economic and environmental importance (Ottosen et al., 2020; Soleimani and Gilbert, 2021). Feed efficiency is generally considered to be stable, but there are significant differences in efficiency among individuals fed the same diet under identical conditions. This variation is largely due to factors that are not well understood. Host genetic variation is a key driver of phenotypic variability (Li et al., 2022; Wang K. et al., 2022). Several studies have previously attempted to elucidate the connections between host genetics, microbiome data, and feed efficiency (Bergamaschi et al., 2020a; Bergamaschi et al., 2020b). The gut microbiome is considered the host’s second genome and is crucial for its host. It provides numerous services, such as nutrient digestion, disease resistance, and the production of vitamins and beneficial metabolites. Additionally, it plays a role in modulating inflammation and stimulating the production of antimicrobial compounds (Broom and Kogut, 2018; Gardiner et al., 2020; Cullen et al., 2022).

Feed efficiency (FE) is a complex trait influenced by feed intake and daily weight gain. Previous studies have demonstrated a correlation between feed efficiency and the gut microbiome. There was no difference in alpha diversity between the HRFI and LRFI groups, consistent with earlier findings (Si et al., 2020). Similar results have been observed in chickens (He et al., 2023). However, in the PCoA analysis comparing the two groups, distinct clustering was observed, though the separation was minimal. This aligns with several studies (McCormack Ursula et al., 2017; Wen et al., 2019; Aliakbari et al., 2021; Chen et al., 2021; Wen et al., 2021). We hypothesize that the minimal structural differences in the gut microbiome could be attributed to the pigs being under the same management, environmental, and nutritional conditions, resulting in only slight variations in the microbial community structure. In this study, the seven most abundant genera were *Prevotella*, *Methylobacterium*, *Campylobacter*, *Phocaeicola*, *Bacteroides, Porphyromonas*, and *Fusobacterium*. *Prevotella* was reported as a core bacterium in fecal samples at 80, 120, and 240 days of age (Ke et al., 2019). Among these, *Prevotella-9* was the first genus to show a significant difference in abundance between the high and low groups, with its abundance being slightly higher in the LRFI group compared to the HRFI group (*p* < 0.05). This finding is consistent with previous research (Si et al., 2020). Certain specific fecal microorganisms may be related to feed efficiency in pigs. The LEfSe analysis revealed that the family *Prevotellaceae* and the genus *Prevotella* were significantly more abundant in LRFI pigs than in HRFI pigs, which is in line with prior studies (Yang et al., 2017).

In this study, the genomic heritability of growth and feed efficiency traits ranged from 0.27 to 0.46, which is consistent with previous findings (Miar et al., 2014; Willson et al., 2020; Li et al., 2022). These traits exhibit moderate to high heritability, indicating that growth and feed efficiency can be improved through genetic selection.

Two SNPs significantly associated with ADG and ADFI were identified. MARC0079871 is an important SNP related to ADG, located near the *CUL2*. Previous studies in mice have shown that *CUL2* can influence the expression of *PRDM16* target genes. The *PRDM16* gene activates brown/beige fat-selective genetic programs and mitochondrial BCAA and fatty acid oxidation while suppressing adipose tissue inflammation and fibrosis (Wang Q. et al., 2022). We speculate that *CUL2* may affect pig growth by indirectly influencing fatty acid oxidation. Additionally, we identified SNP ALGA0000120, located near *RPS6KA2*, in ADFI (Martínez-Montes et al., 2018). Previous research has found that in chickens, RPS6KA2 is associated with benzaldehyde and (E, E)-2,4-decadienal and is involved in the MAPK signaling pathway (Yuan et al., 2022). The MAPK and TGF-*β* signaling pathways interact with the PPAR pathway, regulating lipid metabolism during adipogenesis in chickens, thereby promoting growth (Cui et al., 2012; Ma et al., 2021).

FE is a crucial economic trait that significantly impacts the profitability of livestock industries. Identifying key genes regulating FE through molecular breeding techniques can enhance the efficiency of FE improvement. Previous studies have shown that biological processes such as fat deposition, appetite regulation, and energy metabolism can influence individual feed intake and, consequently, feed efficiency (Silva et al., 2019; Yang et al., 2020; Wu et al., 2021). Nitric oxide has been shown to play a crucial role in systemic metabolic regulation and insulin sensitivity (Jørgensen et al., 2005). It regulates mitochondrial aerobic respiration through mitochondrial activity and oxygen levels (Willens et al., 2014; Saxena et al., 2015). In this study, we identified *ASS1*, an enzyme responsible for mammalian citrulline metabolism. The argininosuccinate produced by *ASS1* is a direct precursor of arginine, which is the primary substrate for intracellular NO synthesis (Pan et al., 2022). Gene *QRFP* significantly activates orexin/hypocretin neurons in the lateral hypothalamus, increasing arousal and appetite behaviors, which leads to increased food consumption (Cook et al., 2022). We hypothesize that such regulation may stimulate appetite, thereby influencing feed intake and affecting feed efficiency Regarding feed conversion ratio, four SNPs associated with this trait were identified, located near the genes *ACSS3*, *AP1S3*, *CUL3*, *MRPL44*, *GLYATL2*, and *LDHB*, as well as the *OR5B21*, *OR5B3*, *OR9I1*, *OR9Q2*. Previous studies have shown that *GLYATL2* facilitates the conjugation of medium-and long-chain fatty acids with glycine, promoting metabolism (Waluk et al., 2010; Xu et al., 2024). *LDHB* catalyzes the interconversion of pyruvate and lactate (Gong et al., 2023; Li R. et al., 2023). It also mediates the conversion of NADH to NAD^+^ in the glycolytic pathway (Li D. et al., 2023), suggesting that *LDHB* might influence feed efficiency by affecting the pyruvate metabolism pathway in pigs. Interestingly, we identified SNPs ASGA0105274 and MARC0067088 near the *OR5B21*, *OR5B3*, *OR9I1*, *OR9Q1*, and *OR9Q2*. These genes belong to the OR family, which is involved in the production of G protein-coupled receptors that detect and transmit olfactory stimuli. Previous research has shown that *OR9Q2* is a key sensor for primary food odors (Haag et al., 2023). It is suggested that the expression of these genes might enhance the pigs’ ability to smell the feed, stimulate their appetite, increase feed intake, and thereby improve feed efficiency.

The estimation of “microbiability” can be used as a tool to quantify the impact of gut microbiota abundance on host phenotypes. Microbiability is defined as the fraction of phenotypic variance that can be inferred from the gut microbiome. This concept was initially proposed by Difford et al. (2016) and Difford et al. (2018) and has been applied to humans (Zhernakova et al., 2024), chickens (Wen et al., 2019), pigs (Yang et al., 2022), sheep (Wang et al., 2023), and cows (Difford et al., 2018; Xue et al., 2020). Aliakbari et al. (2022) found that the proportion of feed efficiency trait variation explained by the gut microbiome is lower than that explained by host genetics. Wen et al. (2021) explored the combined contributions of the gut microbiota and host genetics to feed efficiency in chickens, estimating the microbiability ( $m^{2}$ ) of different gut segments, with fecal $m^{2}$ being 0.01. Déru et al. (2022) investigated the impact of the gut microbiome and host genetics on the digestive and feed efficiency traits of growing pigs fed conventional and high-fiber diets. They used microbial association matrices constructed from different OTU counts and found varying m2 values. In Tang et al. study (Tang et al., 2020), the rectal microbiability for growth and fat deposition traits ranged from 0.09 to 0.11. Our analysis suggests that the level of microbiability is influenced by several factors. Firstly, microbiability varies across different gut segments, with estimates based on fecal samples being lower than those from other segments. Secondly, the method used to construct the microbial similarity matrix also affects the $m^{2}$ (He et al., 2022). Additionally, the filtering criteria for OTUs differ significantly between studies. For example, some researchers retain OTUs present in more than 50% of samples (Difford et al., 2016), while others retain OTUs found in at least 5% of samples (Verschuren et al., 2020).

Although the proportion of trait variance explained by fecal microbiota is small, we still aim to find evidence of host genetic influence on the microbiome. A common approach is to perform joint analysis of genetic loci and gut microbiome abundance. Previous studies have found significant associations between fat deposition, feed efficiency, and specific microbes in the pig gut (He et al., 2016; Fang et al., 2017). However, there is limited research on the impact of host genetics on the abundance of specific microbial taxa in pigs. In our study, we identified 19 genera with significant heritability. The next step is to identify the host genetic variants and genes associated with these heritable microbial taxa. In this study, we conducted a GWAS and identified four SNPs associated with three genera. Previous research has shown that *Trueperella* is associated with inflammation (Sun et al., 2023; Kaura et al., 2024; Liu et al., 2024). However, we identified candidate genes *CCAR2* and *EGR3* associated with *Trueperella*. *Victivallis* is closely associated with obesity and hepatic steatosis (Rodriguez et al., 2020). Studies have shown that inulin treatment in mice fed a high-fat diet revealed a positive correlation between *Victivallis* and liver lipid accumulation and muscle steatosis. We identified eight candidate genes associated with *Victivallis* abundance, with *MYF5*, *MYF6*, *PTPRQ*, and *ACSS3* being the most promising. *MYF5* and *MYF6* are important members of the myogenic regulatory factor family, playing a crucial role in skeletal muscle development and maturation, satellite cell regulation, and muscle regeneration. Interestingly, they are primarily involved in muscle cell differentiation and have a subtle influence on mature muscle (Davegårdh et al., 2017; Zammit, 2017). Pezeshkian et al. (2022) identified *PTPRQ* as a potential key gene influencing feed efficiency in turkeys, involved in phosphatase activity and protein tyrosine phosphatase activity. *ACSS3*, a member of the acyl-CoA synthetase short-chain family, is involved in lipid and carbohydrate metabolism and has been identified as a key enzyme in propionate metabolism (Bidkhori et al., 2018; Zhou et al., 2021; Jia et al., 2022). In cattle, *ACSS3* has been identified as a candidate gene for the C10:0 content in milk fat composition (Buitenhuis et al., 2014). The candidate gene associated with Erysipelatoclostridium is *PITPNB*, a single-domain protein with a hydrophobic cavity that binds phosphatidylinositol (PI) or phosphatidylcholine molecules, playing a role in glycerophospholipid biosynthesis and metabolism (Garner et al., 2012). Considering that certain gut microbes are influenced by host genes, these microbes can be regarded as a host trait from an animal breeding perspective. This underscores the potential of improving pig growth rates and feed conversion ratios by breeding to enhance microbial communities (Benson et al., 2010). Although these studies have revealed important discoveries, there are also limitations. No equally significant SNPs were found in either GWAS or mbGWAS. This phenomenon may be due to the low-density GeneSeek Porcine SNP50K BeadChip, which provides a limited number of SNPs. Another reason might be the small sample size. In future studies, we plan to address this limitation by utilizing higher-density chips for more comprehensive detection and increasing the population size ( Wang et al., 2023; Zhang et al., 2024 ).

Considering the impact of resident gut microbiota on pig growth and feed efficiency, we conducted an in-depth study of microbial taxa significantly associated with these traits. Our research confirmed that *Prevotella_1*, *Lachnospiraceae_UCG-005*, *Prevotella_9*, *Prevotellaceae_NK3B31_group*, *Prevotellaceae_UCG_003*, and *Veillonella* are related to pig growth and feed efficiency, consistent with previous studies. We examined 14 microbes with a detection rate of over 90% and found that *Prevotella_1*, *Prevotellaceae_NK3B31_group*, and *Lachnospiraceae_UCG-005* showed significant positive correlations with ADG, ADFI, and RFI. Our study observed that pigs with higher ADG and ADFI had higher levels of *Prevotella_1*, while those with lower RFI had relatively lower abundances of *Prevotella_1*. This suggests that *Prevotella_1* may enhance the host’s feed utilization efficiency, reflected in phenotypic traits like growth rate. *Prevotella* can ferment various substrates such as starch, peptides, proteins, and hemicellulose, which helps improve the host’s feed efficiency (Ellison et al., 2017; Delgado et al., 2020). By participating in the breakdown and fermentation of complex carbohydrates, *Prevotella* converts starch, cellulose, and other indigestible polysaccharides into more easily absorbable forms for pigs, thereby accelerating growth and improving feed efficiency. Additionally, Prevotella species produce short-chain fatty acids (SCFAs) such as butyrate and propionate during fermentation. These SCFAs are important energy sources for maintaining gut health in pigs, helping to preserve intestinal epithelial integrity and enhance immunity. Weishaar et al. (2020) reported that the abundance of OTUs from the *Lachnospiraceae* and *Prevotellaceae* families significantly impacts FCR and RFI, although the direction of these effects was not specified. Some genera within *Lachnospiraceae* are significantly positively correlated with dietary fiber intake in the pig colon. *Lachnospiraceae* are known to produce enzymes that degrade carbohydrates (Kaoutari et al., 2013). Xu et al. (2022) showed that a long-term high-energy diet (HED) can alter the gut microbiome, reducing the levels of butyrate-producing bacteria, including *Lachnospiraceae*. This indicates that high-energy diets decrease SCFA production. Our findings also highlight the positive role of *Lachnospiraceae*. We observed that pigs with higher ADG and ADFI had a significantly higher relative abundance of *Lachnospiraceae_UCG-005* compared to the low group. This suggests that *Lachnospiraceae* promotes nutrient absorption, leading to increased body weight and feed intake. Bi et al. (2022) conducted an interesting study on weaned piglets. They found that enriching the social environment during lactation helps reduce stress in weaned piglets, significantly increasing the abundance of beneficial gut bacteria like *Prevotella_9*. This, in turn, positively impacts the piglets nutritional metabolism and growth.

## 5 Conclusion

In this study, seven SNPs related to ADG, ADFI, RFI, and FCR were identified. Additionally, six genera *Prevotella_1*, *Lachnospiraceae_UCG-005*, *Prevotella_9*, *Prevotellaceae_NK3B31_group*, *Prevotellaceae_UCG_003*, and *Veillonella* showed correlations with pig growth and feed efficiency. These findings collectively enhance our understanding of the interactions between host genetics and gut microbiota in relation to commercial pig growth and feed efficiency. They may also contribute to developing strategies to improve growth and feed efficiency.

## Data Availability

The data analyzed in this study is subject to the following licenses/restrictions: The datasets used or analyzed during the present study are available from the corresponding author on reasonable request. Reasonable requests to access the dataset can be directed to the corresponding author. Requests to access these datasets should be directed to Zhili Li, pinganzhili@163.com.
