# Supplementary material for: The influence of host genotype and gut microbial interactions on feed efficiency traits in pigs
Source: Front Microbiol. 2024 Nov 13;15:1459773. doi: 10.3389/fmicb.2024.1459773 (PMC11599184; doi:10.3389/fmicb.2024.1459773)
Supplement: Supplementary file 1 [file Supplementary_file_1.docx]

**Supplementary Figures**


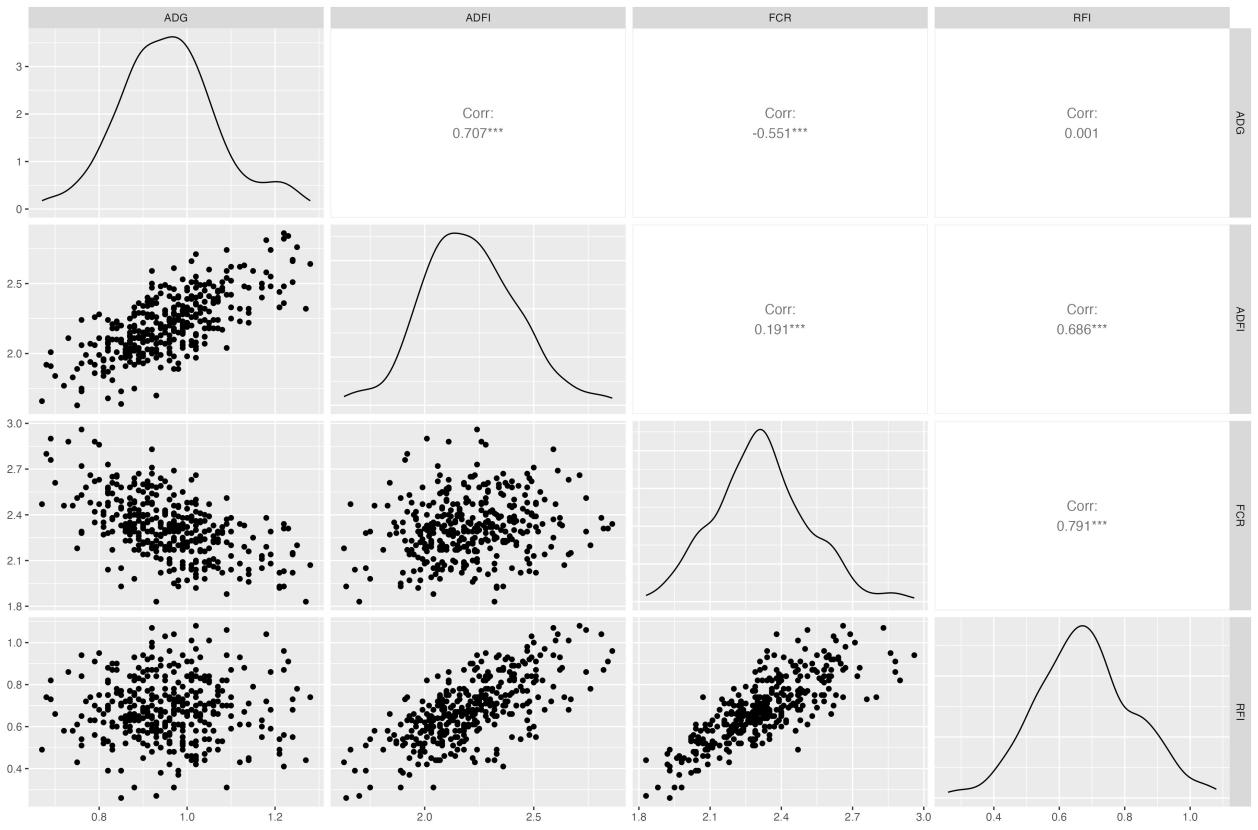


**Supplementary Figure 1. Distribution of growth traits and feed efficiency.** ADFI average daily feed intake; ADG average daily gain; RFI residual feed intake; FCR feed conversion ratio


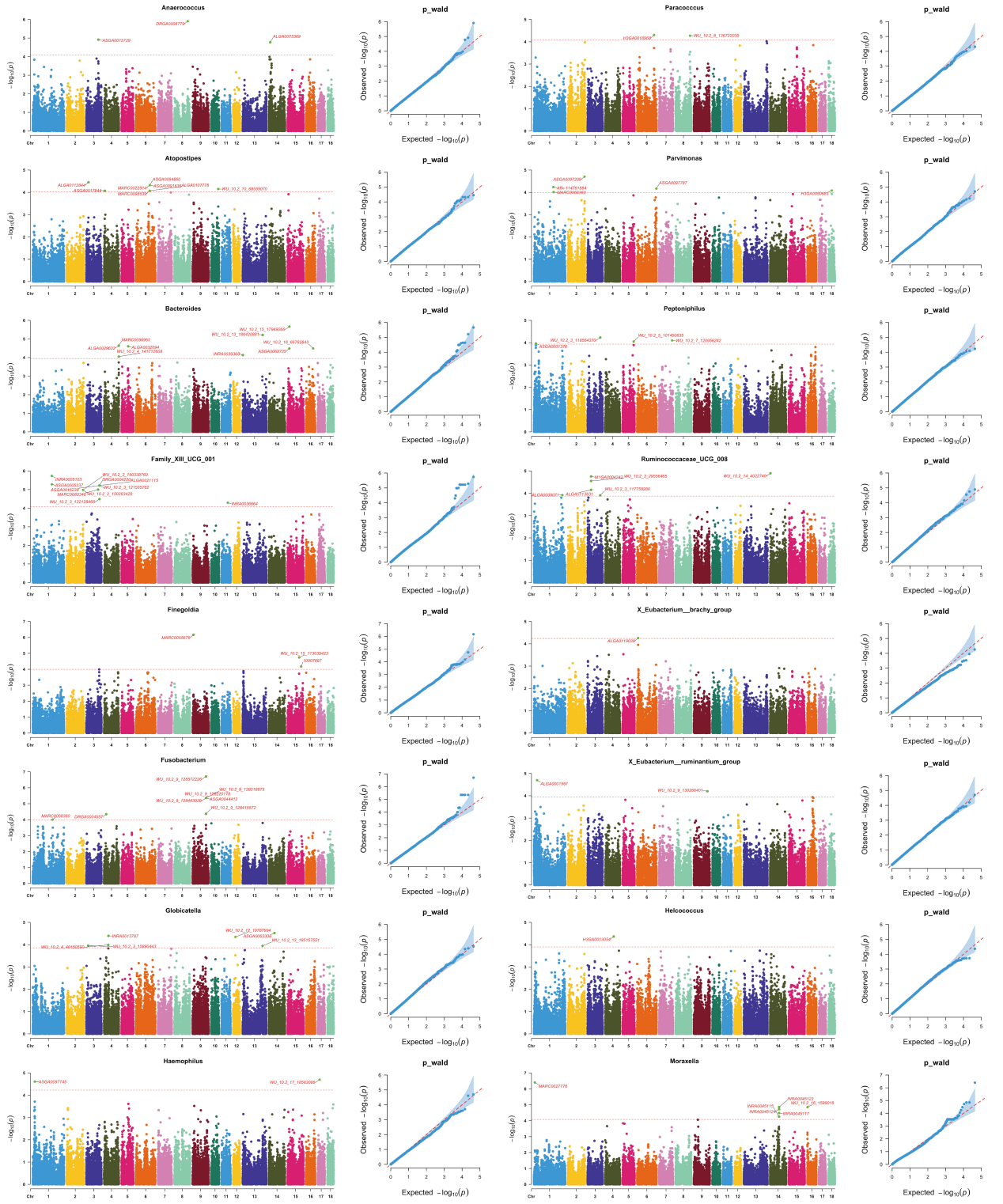


**Supplemental Figure 2. Microbial genome-wide association analysis Manhattan map and Q-Q Plot.**
